# Orthorexia Nervosa and exercise addiction in a sample of Tunisian athlete students

**DOI:** 10.1192/j.eurpsy.2024.841

**Published:** 2024-08-27

**Authors:** N. Smaoui, O. Bouattour, R. Feki, I. Gassara, S. Omri, M. Maalej, L. Zouari, M. Maalej, J. Ben Thabet, N. Charfi

**Affiliations:** psychiatry C department, Hedi chaker University hospital, Sfax, Tunisia

## Abstract

**Introduction:**

Orthorexia and exercise addiction can lead to serious health problems, such as malnutrition and exercise-related injuries.

**Objectives:**

The aims of our study were to assess the prevalence of exercise addiction and orthorexia nervosa in Tunisian students at the Institute of Physical Education in order to investigate the relationship between these different health dimensions.

**Methods:**

An anonymous self-administered questionnaire was distributed to students in the Sfax and Gafsa sports sections during March 2023. The orthorexic tendency was assessed using the ORTO-15 questionnaire. An ORTO-15 score below 40 points indicates orthorexic tendencies. The Exercise Addiction Inventory (EAI) was used to study exercise addiction

**Results:**

In our study, 240 students were included. Mean scores on the ORTO-15 and EAI scales were 38.6 ± 8 and 16.6 ± 4.1 respectively. Participants at risk of exercise addiction had a statistically significant tendency towards orthorexia (p<0.001). Among the students, 82.5% had engaged in regular physical activity at a gym in the last two years. The reasons given by students for going to the gym were muscle strengthening (57.9%) and preparation for a sporting competition (37%).

Among students taking part in sports activities at the gym, the mean ORTHO-15 score was significantly lower among those doing so to prepare for a sports competition (p=0.005). Participants who believed that they were addicted to sport had a statistically greater tendency towards orthorexia (p=0.012).

**Conclusions:**

Our study revealed an association between addictive exercise and orthorexic eating in Tunisian athlete students.

**Disclosure of Interest:**

None Declared

